# LDHA, BIK, and CNIH4 Are Diagnostic Markers of Endoplasmic Reticulum Stress in Lung Cancer Comorbid With Sepsis: Integrating Machine Learning and Single‐Cell Analysis of Immune Signaling

**DOI:** 10.1155/mi/2437852

**Published:** 2026-07-11

**Authors:** Yifeng Pan, Xiaoru Liu, Jiawen Wang, Jing Yan

**Affiliations:** ^1^ The Eighth Clinical Medical College of Guangzhou University of Chinese Medicine, Foshan, 528000, Guangdong, China; ^2^ Foshan Hospital of Traditional Chinese Medicine, Foshan, 528000, Guangdong, China, foshan.gov.cn; ^3^ Graduate School of Guangxi University of Chinese Medicine, Nanning, 530200, Guangxi, China; ^4^ The First Affiliated Hospital of Guangxi University of Chinese Medicine, Nanning, 530200, Guangxi, China, gxtcm.com

**Keywords:** endoplasmic reticulum stress diagnostic markers, immune cell infiltration, lung cancer, sepsis

## Abstract

**Background:**

Lung cancer is intricately associated with the onset of sepsis. Endoplasmic reticulum (ER) stress (ERS) is a cellular stress response to aberrant protein folding in the ER, closely associated with the cellular immune response. Currently, numerous research have elucidated the correlation between ERS and lung cancer, as well as sepsis. The mechanism of ERS in lung cancer comorbid with sepsis requires more investigation.

**Objectives:**

This study aimed to investigate the interacting mechanisms between ERS and the immune response, explore prospective ERS‐related diagnostic biomarkers for lung cancer comorbid with sepsis, and elucidate their underlying pathological roles.

**Methods:**

Datasets for lung cancer and sepsis were sourced from the Gene Expression Omnibus (GEO). Differentially expressed genes (DEGs) and weighted gene coexpression network analysis (WGCNA) modules were intersected with ERS‐related genes. Protein–protein interaction (PPI) and enrichment analyses were conducted. A genetic diagnostic model was developed using exhaustive machine learning algorithms, with accuracy assessed by receiver operating characteristic (ROC) curves and confusion matrices. Hub genes (area under the curve [AUC] ≥ 0.7) were analyzed for immune cell infiltration and cross‐validated using single‐cell RNA sequencing datasets. Crucially, the expression and functional roles of the hub genes were experimentally validated by western blot in clinical tissue cohorts (adjacent normal, lung cancer, and lung cancer with sepsis) and via wound healing and Transwell assays in PC9 lung cancer cells. Finally, prospective therapeutic agents were identified through molecular docking.

**Results:**

Machine learning identified lactate dehydrogenase A (LDHA), Bcl‐2 interacting killer (BIK), and cornichon homolog 4 (CNIH4) as robust diagnostic biomarkers. Western blot analysis confirmed that the protein expression levels of LDHA, BIK, and CNIH4 were significantly upregulated in lung cancer and further elevated in the lung cancer comorbid with sepsis group. In vitro functional assays demonstrated that silencing these genes significantly inhibited the migration and invasion capabilities of PC9 cells. Single‐cell analysis revealed that these markers exhibit cell‐type‐specific expression in malignant cells and regulate immune dysregulation, particularly correlating with the functions of plasma cells and monocytes. Molecular docking indicated that tetrahydro‐NAD and amikacin are promising therapeutic candidates.

**Conclusions:**

We identified and experimentally validated LDHA, BIK, and CNIH4 as specific ERS‐associated diagnostic biomarkers for lung cancer comorbid with sepsis. These markers drive tumor progression and modulate cellular immune responses, providing novel insights and therapeutic targets for this comorbidity.

## 1. Introduction

Lung cancer is a prevalent kind of cancer, mostly comprising small‐cell lung cancer (SCLC) and non‐SCLC (NSCLC). Epidemiological studies [1] indicate that over 2.5 million new lung cancer cases were identified globally in 2022, resulting in over 1.8 million fatalities attributed to the disease; SCLC and NSCLC represent roughly 15% and 85% of lung cancer incidence [2]. Sepsis is a systemic inflammatory response resulting from infection, frequently associated with organ failure, and is a major factor in elevated hospitalization and mortality rates globally [3]. The prevalence of sepsis in intensive care units has been documented at 58 cases per 100,000 person‐years [4], accompanied by a death rate of approximately 41.9% during hospitalization.

An increasing number of research have substantiated that lung cancer is intricately linked to the onset of sepsis. Genetic evidence indicated [5] that lung cancer (including SCLC and NSCLC) considerably elevated the risk of sepsis and its 28‐day death rate. Furthermore, it has been observed that lung cancer is positively connected with the severity of sepsis, and the mortality risk is significantly elevated when both conditions are present [6]. The immunosuppressive characteristics of the tumor microenvironment (TME) are significantly associated with the onset and progression of lung cancer [7]; in addition, the deregulation of immune responses and cytokine storms are critical mechanisms in the pathogenesis of sepsis [8]. The pathophysiology of lung cancer comordid with sepsis remains incompletely elucidated, presenting a significant obstacle to clinical treatment. An comprehensive examination of the pathophysiology of lung cancer comordid with sepsis may be vital in identifying specific treatment therapies.

The endoplasmic reticulum (ER) is a crucial organelle in cells, primarily tasked with protein production and the maintenance of cellular homeostasis. Exposure of cells to stress or inflammation can impair the function of the ER, resulting in the buildup of misfolded or unfolded proteins, a condition referred to as ER stress (ERS) [9]. Numerous studies indicate that ERS is intricately linked to inflammatory responses, and prolonged elevated levels of ERS can initiate intracellular inflammatory cascades, potentially leading to the onset of diseases such as lung cancer and sepsis [10, 11]. In TME, ERS can proficiently modulate immune cell responses [12, 13]. For instance, ERS can influence immunosuppression by modulating antitumor T‐cell responses [14]; ERS‐related proteins can govern the release of ER calcium in macrophages and monocytes, regulating inflammatory and immunological responses in sepsis [15]. However, the role of ERS in lung cancer comordid with sepsis, particularly the distinct impacts of ERS on immune cells, remains ambiguous.

In this study, we utilized an integrative bioinformatics framework and exhaustive machine learning algorithms to identify critical ERS biomarkers associated with the comorbidity of lung cancer and sepsis. CIBERSORT and single‐cell RNA sequencing analyses were employed to elucidate the correlation between these biomarkers and specific immune cell populations. Crucially, to transcend in silico hypothesis generation and directly address the clinical complexity of the comorbid state, we validated the expression of the identified hub genes at the protein level using clinical tissue cohorts specifically comprising patients clinically diagnosed with both lung cancer and sepsis concurrently. Additionally, in vitro functional assays were conducted to confirm the biological impact of these genes on lung cancer progression. Furthermore, molecular docking technology was utilized to suggest prospective treatment agents. This study aimed to discover biomarkers associated with ERS and cellular immunity, elucidate the shared pathophysiology, and provide robust, experimentally validated therapeutic targets for lung cancer comorbid with sepsis.

## 2. Materials and Methods

### 2.1. Data Sources

The Gene Expression Omnibus (GEO) database (http://www.ncbi.nlm.nih.gov/geo) was queried using the keywords “lung cancer” and “sepsis,” and the subsequent criteria for gene expression profiles related to lung cancer and sepsis were established as follows: (i) *Homo sapiens*, (ii) expression profiling by array, (iii) the datasets encompassed both case and control groups, and (iv) the sample size of each group > 5. Thereafter, we acquired datasets pertaining to lung cancer and sepsis. Furthermore, we acquired gene data pertaining to ERS from GeneCards (https://www.genecards.org).

### 2.2. Study Design

**Figure 1:**
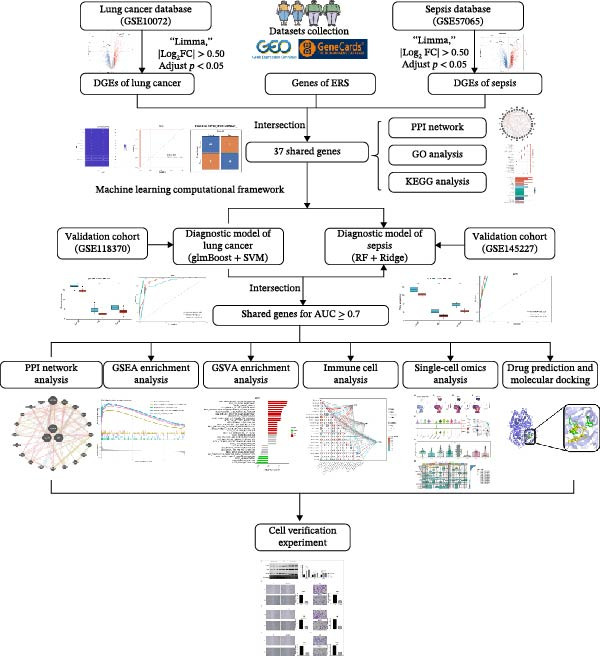
The overall scheme of our investigation comprehensively.

The differentially expressed genes (DEGs) and module genes from weighted gene coexpression network analysis (WGCNA) and ERS genes related to lung cancer and sepsis were intersected. The interaction module genes were utilized for protein–protein interaction (PPI) network analysis, GO enrichment analysis, and KEGG enrichment analysis. Additionally, gene diagnosis models were developed using machine learning, and receiver operating characteristic (ROC) curves and confusion matrices were generated to evaluate the accuracy of the diagnostic model genes. Then, the diagnostic model genes of lung cancer and sepsis were intersected to identify hub genes with an area under the curve (AUC) ≥ 0.7. These genes underwent PPI network analysis, gene set variation analysis (GSVA), gene set enrichment analysis (GSEA), and immune cell infiltration analysis, thereby elucidating the molecular mechanisms underlying lung cancer comordid with sepsis. Ultimately, prospective therapeutic agents for lung cancer comorbid with sepsis were forecasted by molecular docking based on these hub genes. The data utilized in this study were sourced from public databases, thereby negating the necessity for reapplication for clinical ethics approval. Figure 1 delineates the overall scheme of our investigation comprehensively.

### 2.3. Screening of DEGs

The gene data related to lung cancer and sepsis were normalized utilizing the R software package including “limma” and “normalizeBetweenArrays” [16]. The DEGs of lung cancer and sepsis were individually identified, and volcano plots and heatmaps of the DEGs were generated using the R software package"pheatmap” and “ggplot2.” The thresholds for the DEGs were established at *p* < 0.05 and |log2FC| > 0.50. DEGs with a log2FC over 0.50 were classified as upregulated genes, and those below −0.50 were categorized as downregulated genes.

### 2.4. Screening of WGCNA Gene Modules

WGCNA is an essential instrument for identifying genes associated with lung cancer and sepsis [17]. The absent data were evaluated using “goodSamplesGenes,” and genes exhibiting low expression variability were removed by “cutreeStatic.” The weighted adjacency matrices were produced using the power function, and the best soft threshold (*β*) was determined by the “pickSoftThreshold” function. The adjacency matrices were converted into topological overlap matrices (TOMs), and the corresponding 1−TOM dissimilarities were calculated using “TOMsimilarity.” Hierarchical clustering was performed using group average linkage, leading to the consolidation of genes with similar expression patterns into gene modules, with a minimum threshold of 60 genes per module set to enable the creation of a gene dendrogram. The diversity of module feature genes was evaluated, and the shear height of the module dendrogram was set at 0.25, leading to the consolidation of certain modules for subsequent analyses.

### 2.5. Enrichment Analysis

The DEGs from lung cancer and sepsis, the gene modules derived by WGCNA, and ERS genes were intersected, and a Venn diagram of the intersected module genes was generated using “ggvenn.” The intersected modular genes were uploaded to the GeneMANIA website (https://genemania.org) for PPI network analysis to evaluate gene interactions. GO functional annotation and KEGG enrichment analysis were conducted utilizing “clusterProfiler” and “enrichplot.” A *p*‐value of less than 0.05 indicates the statistical significance of the results.

### 2.6. Machine Learning

We employed different machine learning methods, including Support Vector Machine (SVM) [18], Random Forest (RF) [19], Glmboost [20], and Ridge [21], to construct a model for predicting lung cancer associated with sepsis based on the intersection module genes. To gain a comprehensive grasp of these models’ performance, we assessed and compared their efficacy utilizing the “survcomp” software package. The GSE10072 dataset of lung cancer served as the training set, while the GSE118370 dataset functioned as the test set; the GSE57065 dataset of sepsis served as the training set, while the GSE145227 dataset functioned as the test set. The optimal model was determined by averaging its performance throughout the training and test sets, the ROC curve was generated utilizing the “pROC” package, and the confusion matrix was visualized with “ggplot2.” Our experimental methods were adapted from those used in previous related studies [22].

### 2.7. Hub Gene Evaluation and Enrichment Analysis

The optimal model genes for lung cancer and sepsis were intersected and illustrated in a Venn diagram. The “ggpubr” and “Performance Analytics” packages were utilized to generate box plots and correlation graphs for the intersected model genes. To assess the potential utility of intersection model genes in predicting lung cancer comorbid with sepsis, we generated ROC curves and computed the corresponding AUC. Intersection model genes with an AUC ≥ 0.7 were designated as hub genes, deemed to possess significant clinical value. The hub genes were examined by PPI network analysis utilizing the GeneMANIA website, while R software packages “org.Hs.eg.db,” “clusterProfiler,” “GSEABase,” and “GSVA” were employed to assess the enrichment of hub genes.

### 2.8. Immune Cell Infiltration Analysis

The immune cell infiltration of lung cancer and sepsis was assessed using “CIBERSORT,” with a significance threshold of *p* < 0.05. The invasion of immune cells was examined and depicted utilizing R software packages “reshape2” and “ggpubr.” The relationship between hub genes of lung cancer and sepsis, as well as immune‐infiltrating cells, was examined utilizing R software packages “tidyverse” and “linkET.” Pearson’s correlation coefficient was employed to ascertain the relationship between hub genes and immune‐infiltrating cells, facilitating the identification of genes and immune cells with possible biological relevance in lung cancer and sepsis.

### 2.9. Drug Prediction and Molecular Docking

Molecular docking technology is essential for investigating protein–drug interactions and has emerged as a significant catalyst in the drug development process [22]. To identify prospective therapeutic options for lung cancer comorbid with sepsis, an extensive molecular docking analysis was conducted on medicines that interact with hub genes. Initially, chemicals associated with the hub gene were examined in Drugbank (https://go.drugbank.com) or DGIdb (https://dgidb.org). The 3D structure files (sdf format) of these compounds were acquired from PubChem (https://pubchem.ncbi.nlm.nih.gov) and subsequently transformed into “mol2” format files using the Chem3D software (version 22.0.0). Concurrently, the protein structure data (PDB format) associated with hub genes were acquired from PDB (https://www.rcsb.org) to serve as receptors for molecular docking. Afterwards, AutoDockTools software (version 1.5.7) was utilized to dehydrate and hydrogenate the receptor and ligand to ensure the precision of molecular docking. Molecular docking was subsequently conducted using AutoDock Vina to identify the optimal binding mode according to the binding energy. The molecular docking data were visualized using Pymol software (version 3.1.3) to produce three‐dimensional images of the protein–drug interactions. The virtual screening pipeline filtered therapeutic candidates based on their binding affinity, with a binding energy threshold strictly set at < −5.0 kcal/mol to indicate strong and stable conformation interactions.

### 2.10. Clinical Specimen Collection and Grouping

Our study involved three specific patient cohorts: tumor‐adjacent normal tissues (Adj‐Normal), lung cancer tissues (LC), and tissues from patients with both lung cancer and sepsis (LC + Sepsis). We made sure to freeze these specimens at −80°C immediately after they were taken during surgery or by biopsy to keep them ready for later tests. The ethics team at the First Affiliated Hospital of Guangxi University of Chinese Medicine gave their formal go‐ahead (Number AF/SC‐08/04.0), and we confirmed that every participant signed a written consent form before we began.

### 2.11. Cell Culture and Transfection

The human lung cancer cell line PC9 was obtained from the Cell Bank of the Chinese Academy of Sciences (Shanghai, China). Cells were maintained in RPMI‐1640 medium supplemented with 10% fetal bovine serum (FBS) at 37°C in a humidified 5% CO_2_ atmosphere. To knock down the target genes, short hairpin RNA (shRNA) sequences targeting lactate dehydrogenase A (LDHA), Bcl‐2 interacting killer (BIK), and cornichon homolog 4 (CNIH4), along with a negative control (sh‐NC), were cloned into a vector (the target sequences and vector construction were performed by Guanghou All‐Perfect Biological Technolocy Co., Ltd.). When cell confluence reached 50%–70%, transfection was performed using Lipofectamine 3000. Cells were collected 48 h after transfection for subsequent experiments.

### 2.12. Western Blot Analysis

Total protein was isolated from tissues and cells using RIPA buffer supplemented with protease inhibitors, quantified with a BCA assay, and then 30 μg aliquots were resolved by SDS‐PAGE and transferred to PVDF membranes. The membranes were blocked with 5% nonfat milk for 2 h at room temperature and then incubated overnight at 4°C with primary antibodies against LDHA (Affinity, DF6280, 1:1000), BIK (Affinity, AF6428, 1:1000), CNIH4(Abmart, PH5782, 1:1000), and beta‐actin (Affinity, AF7018, 1:1000). On the following day, HRP‐conjugated secondary antibodies (Affinity, S0001, 1:5000) were added and incubated for 1 h at room temperature. Protein bands were visualized with an ECL detection system and quantified by densitometric analysis in ImageJ.

### 2.13. Wound Healing Assay

Transfected PC9 cells were plated in 6‐well plates and grown to 80% confluent. A straight scratch wound was introduced using a 200 μL pipette tip. The monolayer was then rinsed with PBS to remove debris and incubated with a serum‐free medium. Wound images were captured at 0 and 6 h under a microscope, and the scratch width was measured. The migration rate was calculated as: (width at 0 h–width at 6 h) / width at 0 h × 100%.

### 2.14. Transwell Invasion Assay

The invasive capacity of the cells was measured with Matrigel‐precoated Transwell inserts. Briefly, 5 × 10^4^ transfected PC9 cells resuspended in 200 μL of serum‐free medium were added to the upper compartment, while 600 μL of complete medium containing 10% FBS served as the chemoattractant in the lower compartment. Following a 24 h incubation, the cells that had invaded through the Matrigel were fixed (4% paraformaldehyde), stained (0.1% crystal violet), and quantified in five randomly selected fields under a light microscope.

### 2.15. Statistical Analysis

All bioinformatics processing and statistical analyses were conducted using R software (version 4.3.3). The Wilcoxon rank‐sum test was employed to compare continuous variables between two independent groups, while the Kruskal–Wallis test was used to evaluate AUCell score differences across multiple cell types in the single‐cell dataset. Pearson’s correlation analysis was utilized to assess the global sample integrity and the associations between hub genes and immune‐cell infiltration. For clinical tissue evaluations and in vitro functional assays, statistical analyses and graphing were performed using GraphPad Prism 9.0. Quantitative data were presented as the mean ± standard deviation (SD). Differences between two experimental groups were analyzed using the independent‐samples *t*‐test, whereas comparisons among three or more groups were performed using a one‐way analysis of variance (ANOVA) followed by Tukey’s post hoc test. Prior to ANOVA, data were assessed for normal distribution and homogeneity of variance using the Shapiro–Wilk and Levene’s tests, respectively. Throughout the study, a two‐tailed *p* < 0.05 was considered statistically significant ( ^∗^ *p* < 0.05,  ^∗∗^ *p* < 0.01, and  ^∗∗∗^ *p* < 0.001). The statistical framework was adapted from previously established methodologies [22].

## 3. Results

### 3.1. Expression Analysis of DEGs in Lung Cancer and Sepsis

Lung cancer obtained two datasets (GSE10072 and GSE118370), while sepsis obtained two datasets (GSE57065 and GSE145227); Table 1 shows specifics. A total of 1800 DEGs were identified in the lung cancer dataset, comprising 965 upregulated genes and 835 downregulated genes (Supporting Information 1: Table S1A). A total of 3317 DEGs were identified in the sepsis dataset, comprising 1819 upregulated genes and 1498 downregulated genes (Supporting Information 2: Table S1B). Heatmaps and volcano plots illustrated the top 100 DEGs and the top 20 DEGs in lung cancer and sepsis (Figure 2A,B).

**Table 1:** Detailed information of the datasets.

GSE series	Disease	Country	Platform file name	Number of cases samples	Number of control samples
GSE10072	Lung cancer	USA	GPL96	58	49
GSE118370	Lung cancer	China	GPL570	6	6
GSE57065	Sepsis	France	GPL570	82	25
GSE145227	Sepsis	China	GPL23178	10	12

Figure 2Expression analysis of DEGs in lung cancer and sepsis. (A) Heatmap and volcano plot of DEGs in lung cancer. (B) Heatmap and volcano plot of DEGs in sepsis.

**Figure:**
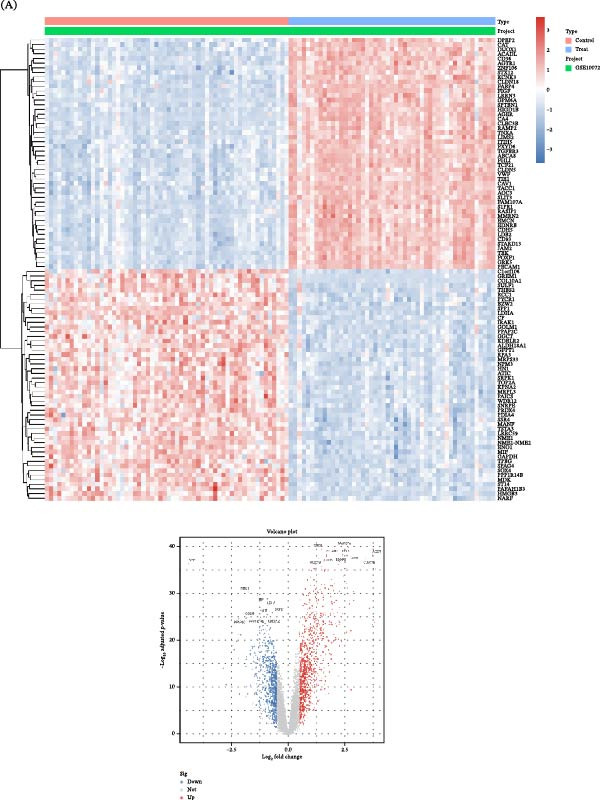

**Figure:**
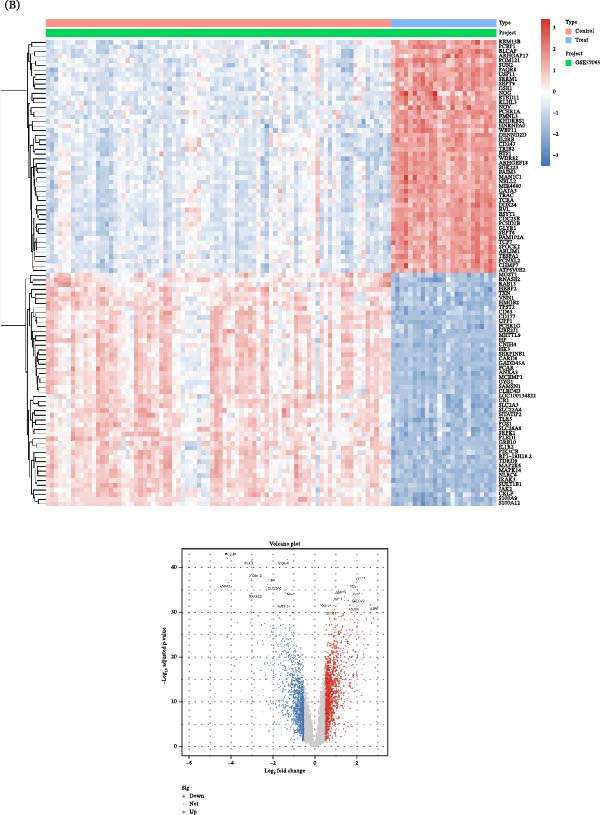

### 3.2. WGCNA Construction and Gene Module Screening

The WGCNA algorithm was employed to develop gene modules specifically associated with lung cancer and sepsis (Supporting Information 3 and 4: Table S2A,S2B). A scale‐free network was established in lung cancer with *β* = 4 and *R* ^2^ = 0.8 (Figure 3A,B). Five gene modules were eventually obtained by the construction of a cluster dendrogram and dynamic tree‐cutting (Figure 3C). The modules were illustrated using a heatmap (Figure 3D). The findings revealed that the MEturquoise module (cor = 0.95; *p* = 2e–54) demonstrated a substantial positive correlation with lung cancer, while the MEblue module (cor = −0.72; *p* = 1e–18) exhibited a significant negative association with lung cancer. The MEturquoise module comprised 1549 genes (Supporting Information 3: Table S2A).

Figure 3WGCNA screening for significant gene modules. (A,B) In lung cancer, *β* = 4 is the optimal soft threshold based on the results of scale independence and average connectivity. (C) Various colors under the gene dendrogram showing gene coexpression modules in lung cancer. (D) Heatmap of the association between modules and lung cancer. (E,F) In sepsis, *β* = 6 is the optimal soft threshold based on the results of scale independence and average connectivity. (G) Various colors under the gene dendrogram showing gene coexpression modules in sepsis. (H) Heatmap of the association between modules and sepsis. Correlation coefficients (cor) and *p*‐values are separately indicated by numbers in parentheses.

**Figure:**
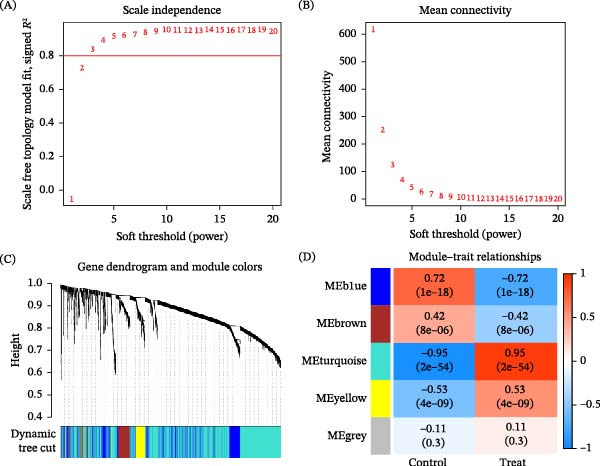

**Figure:**
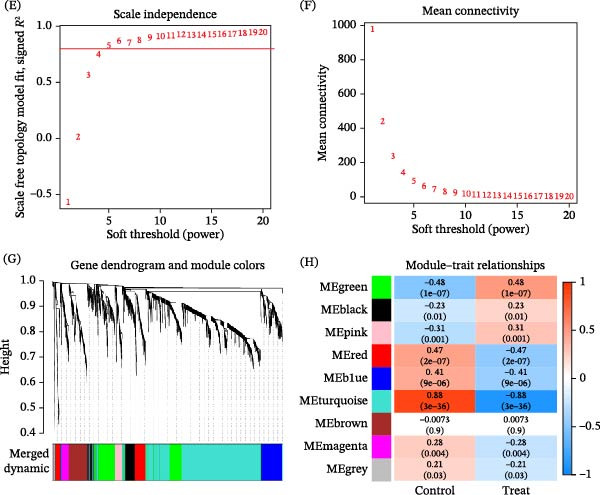

A scale‐free network was established in sepsis when *β* = 6 and 0.5 < R2 < 1.0 (Figure 3E,F). Nine gene modules were eventually obtained by the construction of a cluster dendrogram and dynamic tree cutting (Figure 3G). The modules were illustrated using a heatmap (Figure 3H). The findings revealed that the MEturquoise module (cor = −0.88; *p* = 3e–36) exhibited a substantial negative connection with sepsis, while the MEgreen module (cor = 0.48; *p* = 1e–07) demonstrated a significant positive association with sepsis. The MEturquoise module comprised 1494 genes (Supporting Information 4: Table S2B).

### 3.3. Enrichment Analysis of Intersection Module Genes

The intersections of DEGs and WGCNA gene modules for lung cancer and sepsis were obtained, respectively. Then, the intersecting genes of both lung cancer and sepsis were intersected with ERS genes (relevance score > 5) again, obtaining a total of 37 intersecting module genes (Figure 4A). The interactions among these intersecting modular genes were illustrated by the PPI network diagram (Figure 4B), encompassing 20 associated genes and a total of 556 edges. GO enrichment analysis identified 1881 GO keywords (Supporting Information 5: Table S3). There are 1519 terms associated with biological processes (BPs), such as ameboidal‐type cell migration (GO:0001667), regulation of epithelial cell migration (GO:0010634), and epithelial cell migration (GO:0010632); 143 terms associated with cellular components (CCs), including apical plasma membrane (GO:0016324), apical part of the cell (GO:0045177), and ER lumen (GO:0005788); and 219 terms associated with molecular functions (MFs), such as steroid binding (GO:0005496), lipid transporter activity (GO:0005319), and protein‐membrane adaptor activity (GO:0043495) (Figure 4C,D).

Figure 4Enrichment analysis of DEGs and WGCNA intersection module genes. (A) Venn diagram of the intersection of DEGs, WGCNA gene modules, and ERS genes in lung cancer and sepsis. (B) PPI network diagram of the intersection module genes. (C) Bubble chart of the GO enrichment analysis results of the intersection module genes. (D) Circle map of the GO enrichment analysis results of the intersection module genes. (E) Bar chart of the KEGG enrichment analysis results of the intersection module genes.

**Figure:**
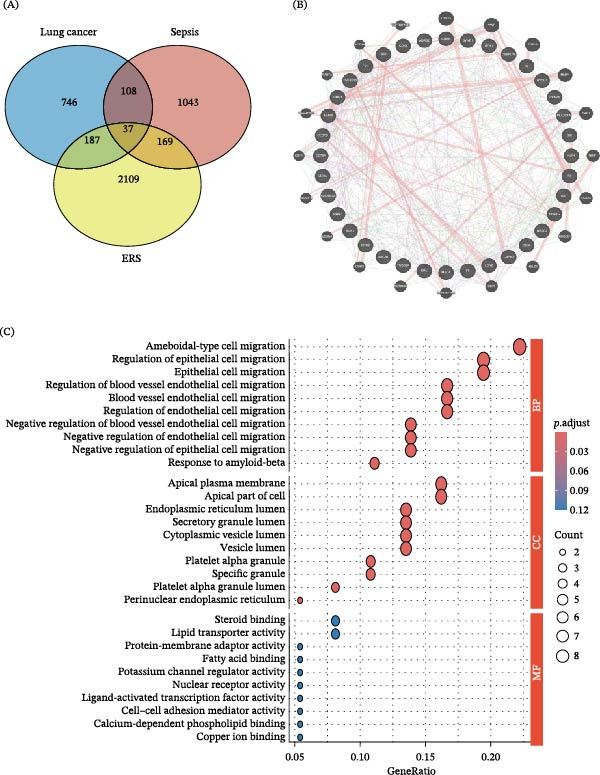

**Figure:**
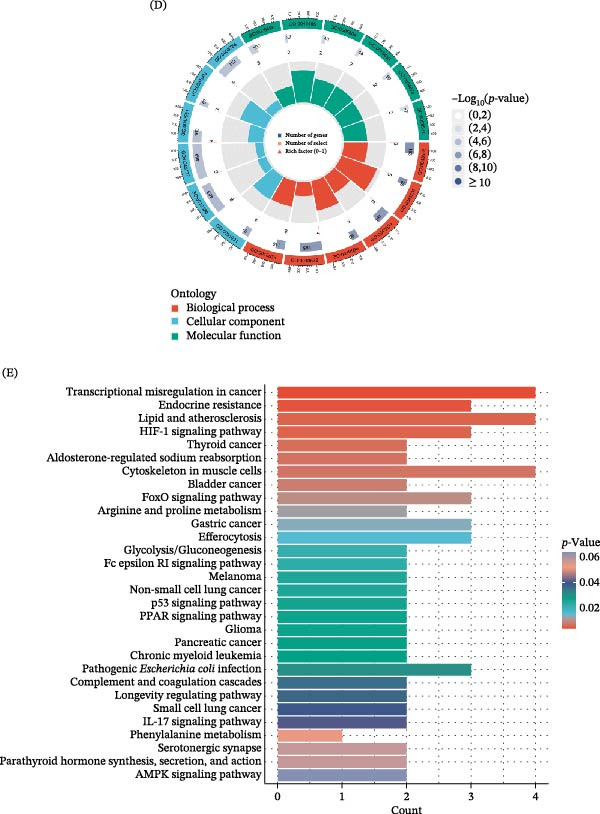

KEGG analysis identified 150 enriched pathways (Supporting Information 6: Table S4). The bar charts indicated that the intersection model genes were mainly engaged in pathways including the HIF‐1 signaling pathway, p53 signaling pathway, and Fc epsilon RI signaling pathway (Figure 4E). In conclusion, we found that ERS can influence the vital physiopathology of lung cancer comorbid with sepsis via various BPs and signaling pathways.

### 3.4. Machine Learning

**Figure 5:**
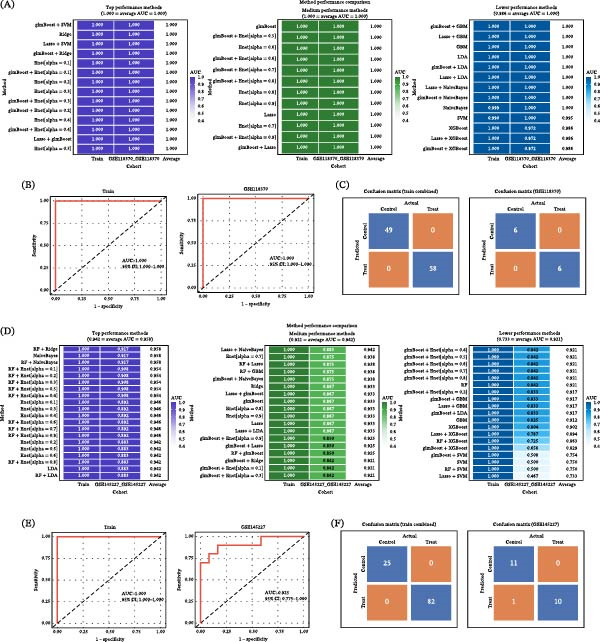
Results of machine learning algorithm. (A) Results of 39 machine learning algorithms for lung cancer. (B) ROC curves of the “glmBoost+SVM” algorithm for the lung cancer training set and test set. (C) Confusion matrices of the “ glmBoost+SVM” algorithm for the lung cancer training set and test set. (D) Results of 57 machine learning algorithms for sepsis. (E) ROC curves of the “RF+Ridge” algorithm for the sepsis training set and test set. (F) Confusion matrices of the “RF+Ridge” algorithm for the sepsis training set and test set.

A total of 39 machine learning algorithms were identified in lung cancer, with “glmBoost+SVM,” “Ridge,” and “Lasso+SVM” ranking as the top three algorithms by average AUC (Figure 5A). The ROC curves and confusion matrices were generated using these three algorithms, revealing that the model genes of “glmBoost+SVM” achieved an AUC of 1.000 in both the training set (GSE10072) and the test set (GSE118370), along with a high accuracy rate (Figure 5B,C). A total of 57 machine learning algorithms were identified in sepsis, with “RF + Ridge,” “NaiveBayes,” and “RF + NaiveBayes” ranging as the top three algorithms by average AUC (Figure 5D). The ROC curves and confusion matrices were generated using these three algorithms, revealing that the model genes of “RF + Ridge” achieved an AUC of 1.000 in training set (GSE57065) and 0.925 in the test set (GSE145227), with the confusion matrices demonstrating a high accuracy (Figure 5E,F).

### 3.5. Evaluation of Hub Gene

**Figure 6:**
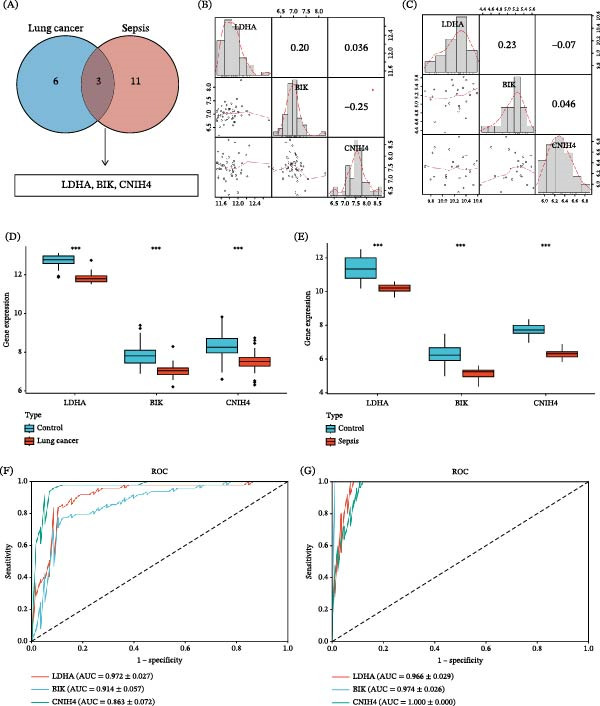
Evaluation of hub gene. (A) Intersection Venn diagram of lung cancer model genes and sepsis model genes. (B) Correlation plot of intersecting model genes in lung cancer. (C) Correlation plot of intersecting model genes in sepsis. (D) Box plot of intersecting model genes in lung cancer. (E) Box plot of intersecting model genes in sepsis. (F) ROC curve of hub genes in lung cancer. (G) ROC curve of hub genes in sepsis.  ^∗^ *p* < 0.05,  ^∗∗^ *p* < 0.01,  ^∗∗∗^ *p* < 0.001.

The lung cancer model genes from the “glmBoost+SVM” algorithm and the sepsis model genes from the “RF + Ridge” algorithm were intersected, resulting in the identification of three common model genes (Figure 6A). Figure 6B,C illustrate the association among these three intersection model genes in lung cancer and sepsis. Box plots demonstrated that LDHA, BIK, and CNIH4 were downregulated in both lung cancer and sepsis models (Figure 6D,E). ROC curves demonstrated that the AUC of LDHA, BIK, and CNIH4 was ≥0.7 in both lung cancer and sepsis (Figure 6F,G), suggesting that these three hub genes may be important to the pathophysiology of both conditions.

### 3.6. Hub Gene Enrichment Analysis

The PPI network diagram illustrated the interaction relationships among three hub genes: LDHA, BIK, and CNIH4, comprising three defined genes, 20 associated genes, and 245 edges (Figure 7A). The GSEA results indicated that, in the lung cancer dataset, LDHA was primarily associated with BPs including the p53 signaling pathway, Nod‐like receptor signaling pathway, and Toll‐like receptor signaling pathway; BIK was implicated in significant processes such as the Wnt signaling pathway, pathways in cancer, and primary immunodeficiency; CNIH4 was closely linked to the calcium signaling pathway, PPAR signaling pathway, MAPK signaling pathway, and T‐cell receptor signaling pathway (Figure 7B and Supporting Information 7: Table S5). In the sepsis dataset, LDHA was primarily associated with BPs, including chemokine signaling pathway, MAPK signaling pathway, JAK/STAT signaling pathway, and Notch signaling pathway; BIK was implicated in significant processes including the chemokine signaling pathway, NOD‐like receptor signaling pathway, and B‐cell receptor signaling pathway; and CNIH4 was closely linked to primary immunodeficiency, calcium signaling pathway, and Wnt signaling pathway (Figure 7C, Supporting Information 7: Table S5).

Figure 7PPI network analysis and enrichment analyses. (A) PPI network of the three hub genes constructed by GeneMANIA. (B) GSEA of LDHA, BIK, and CNIH4 in lung cancer datasets. (C) GSEA of LDHA, BIK, and CNIH4 in sepsis datasets. (D) GSVA of LDHA, BIK, and CNIH4 in lung cancer datasets. (E) GSVA of LDHA, BIK, and CNIH4 in sepsis datasets.

**Figure:**
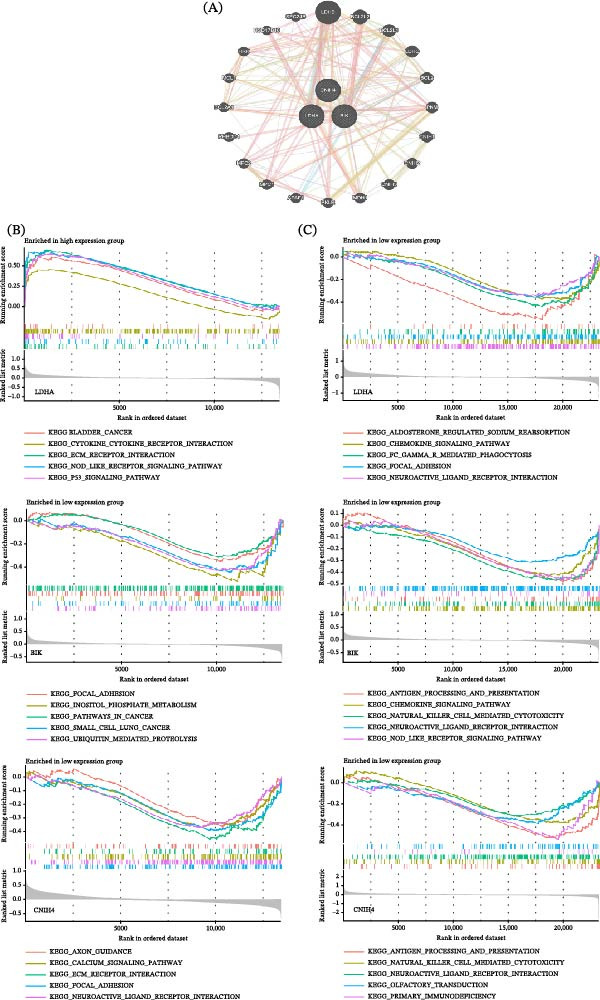

**Figure:**
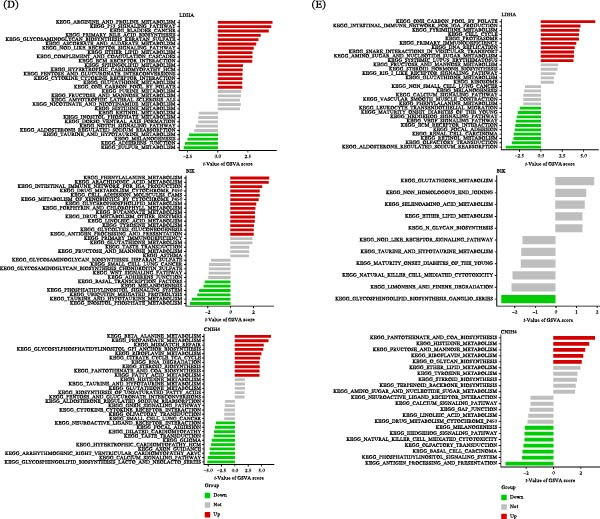

GSVA results indicated (Figure 7D,E, Supporting Information 8: Table S6) that in the lung cancer and sepsis datasets, LDHA was associated with fructose and mannose metabolism, glutathione metabolism, and ECM receptor interaction; BIK was linked to taurine and hypotaurine metabolism and glutathione metabolism; and CNIH4 was linked to histidine metabolism, pantothenate and CoA biosynthesis, and the calcium signaling pathway.

GSEA indicated that hub genes are involved in significant pathogenic processes of lung cancer and sepsis via immune‐related pathways, including the Nod‐like receptor signaling pathway, MAPK signaling pathway, and Wnt signaling pathway. CNIH4 participated in the onset and progression of lung cancer via the T‐cell receptor signaling pathway, whereas LDHA was implicated in the critical pathophysiology of sepsis through the B‐cell receptor signaling pathway. The findings indicated that these three hub genes may influence the pathogenic mechanisms of lung cancer and sepsis through the modulation of the immune system.

### 3.7. Immune Cell Infiltration Analysis

In the lung cancer dataset, the expression levels of negative B cells, plasma cells, T follicular helper cells, regulatory T cells (Tregs), gamma delta T cells, M1 macrophages, and resting dendritic cells significantly decreased, whereas the expression levels of CD8+ T cells, resting NK cells, monocytes, M2 macrophages, resting mast cells, and neutrophils significantly increased (Figure 8A). Negative B cells were negatively correlated with monocytes; plasma cells were negatively correlated with M0 macrophages; CD8+ T cells were negatively correlated with resting CD4+ memory T cells; resting NK cells were negatively correlated with M1 macrophages; M2 macrophages were negatively correlated with activated dendritic cells; resting mast cells were negatively correlated with activated mast cells; T follicular helper cells were positively correlated with M1 macrophages; gamma delta T cells were positively correlated with M1 macrophages; resting dendritic cells were positively correlated with M0 macrophages; and neutrophils were positively correlated with resting NK cells (Figure 8B). Figure 8C illustrates that LDHA were not only negatively correlated with CD8+ T cells, Tregs, activated NK cells, and monocytes,but also demonstrated a positive correlation with plasma cells, resting CD4+ memory T cells, activated CD4+ memory T cells, and activated mast cells. Additionally, CNIH4 displayed a negative correlation with CD8+ T cells and activated mast cells, alongside a positive correlation with resting NK cells, M0 macrophages, resting mast cells, eosinophils, and neutrophils.

Figure 8Immune cell infiltration analysis. (A) Box plot of immune cell infiltration in lung cancer. (B) Heatmap of immune cell infiltration in lung cancer. (C) Correlation analysis of three hub genes with immune cells in lung cancer. (D) Box plot of immune cell infiltration in sepsis. (E) Heatmap of immune cell infiltration in sepsis. (F) Correlation analysis of three hub genes with immune cells in sepsis.  ^∗^ indicates significance level ( ^∗^ *p* < 0.05,  ^∗∗^ *p* < 0.01, and  ^∗∗∗^ *p* < 0.001).

**Figure:**
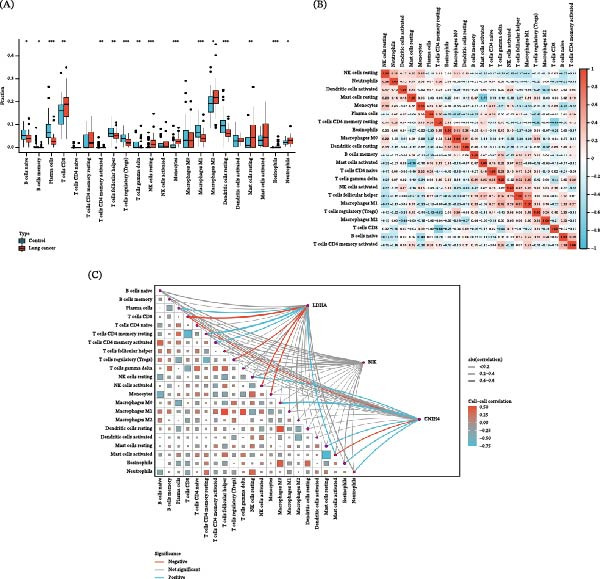

**Figure:**
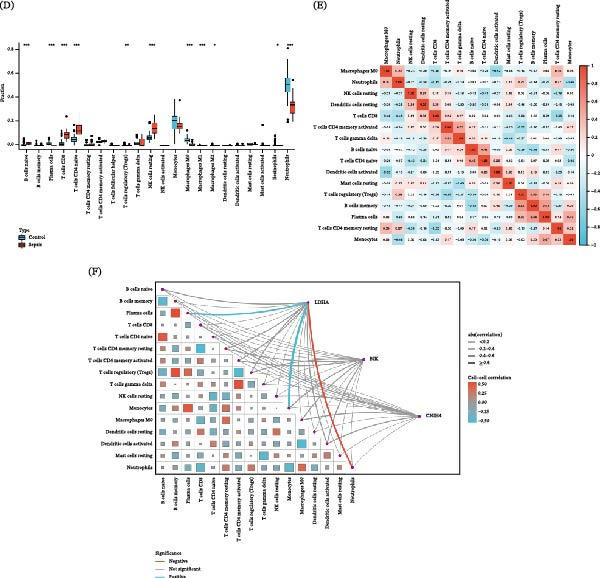

In the sepsis dataset, the expression levels of naive B cells, CD8+ T cells, naive CD4+ T cells, and resting NK cells were significantly raised, whereas the expression levels of plasma cells, M0 macrophages, and neutrophils were significantly decreased (Figure 8D). Naive B cells exhibited a positive correlation with naive CD4+ T cells and a negative correlation with memory B cells; CD8+ T cells negatively correlated with resting CD4+ memory T cells; resting NK cells were negatively correlated with naive CD4+ T cells; plasma cells were positively correlated with memory B cells; M0 macrophages were negatively correlated with activated dendritic cells; and neutrophils were negatively correlated with monocytes (Figure 8E). Figure 8F illustrates that LDHA exhibited a negative correlation with neutrophils and a positive correlation with plasma cells and monocytes. The results indicated that these hub genes may significantly influence the cellular immune response, particularly LDHA, which may be involved in the vital pathogenesis of lung cancer comorbid with sepsis via immune cells such as plasma cells and monocytes.

### 3.8. Single‐Cell Dataset Reveals Immune Cell Specific Expression of ERS Markers LDHA, BIK, and CNIH4 in NSCLC.

To dissect the cell‐type‐specific expression patterns of ERS markers LDHA, BIK, and CNIH4 and their association with immune regulation in NSCLC, we performed single‐cell RNA‐seq analysis on the GSE143423 dataset and cross‐validated findings across 10 independent NSCLC cohorts. First, we annotated major cell populations from the GSE143423 dataset, encompassing immune cells, epithelial/malignant cells, and stromal cells (Figure 9A). The nebula map shows LDHA, BIK, and CNIH4 gene expression levels, which are mainly expressed in malignant cells (Figure 9B–D): These cell‐type‐specific expression patterns were consistent with our prior bulk RNA‐seq and immune infiltration analyses. Next, we analyzed the correlation between the marker expression and immune cell function (Figure 9E). We calculated the correlation between all cells and LDHA, BIK, and CNIH4 gene set correlation; LDHA, BIK, and CNIH4 are positively correlated with tumor cells and negatively correlated with immune cells (Figure 9F). Violin plot displayed the expression differences of the LDHA, BIK, and CNIH4 gene set AUCell scores across different cell types (Kruskal test, *p* < 0.05). Cross‐validation across 10 NSCLC datasets confirmed the robust diagnostic performance of the LDHA, BIK, and CNIH4 panels (Figure 9H). These single‐cell analyses demonstrate that LDHA, BIK, and CNIH4 exhibit cell‐type‐specific expression patterns in NSCLC, respectively. Their expression correlates with key immune cell functional markers, providing direct evidence that these ERS markers modulate immune responses through cell‐specific mechanisms. Cross‐dataset validation further reinforces their potential as stable diagnostic biomarkers for NSCLC, laying the foundation for understanding their role in lung cancer comorbid with sepsis.

Figure 9Single‐cell dataset reveals immune cell specific expression of ERS markers LDHA, BIK, and CNIH4 in NSCLC. (A) Distribution of single‐cell data after UMAP dimensionality reduction (GSE143423). (B–D) The nebula map shows LDHA, BIK, and CNIH4 gene expression levels, with red indicating higher expression and gray indicating lower expression. (E) Heatmap of *Z*‐scores representing the relative expression of LDHA, BIK, and CNIH4 across all annotated cell populations in GSE143423. (F) Calculated the correlation between all cell contents and LDHA, BIK, and CNIH4 gene set AUC scores using Spearman correlation analysis, a positive correlation (red), and blue represents a negative correlation. (G) Assessing the expression differences of the LDHA, BIK, and CNIH4 gene set AUCell scores across different cell types (Kruskal test, *p* < 0.05). (H) The heatmap shows that multiomics data analysis reveals that LDHA, BIK, and CNIH4 gene expression exhibits immune cell heterogeneity. A total of 10 independent NSCLC datasets (GSE99254, GSE117570, GSE127465, GSE131907, GSE139555, GSE143423, GSE146100, GSE150660, GSE151537, and GSE153935).

**Figure:**
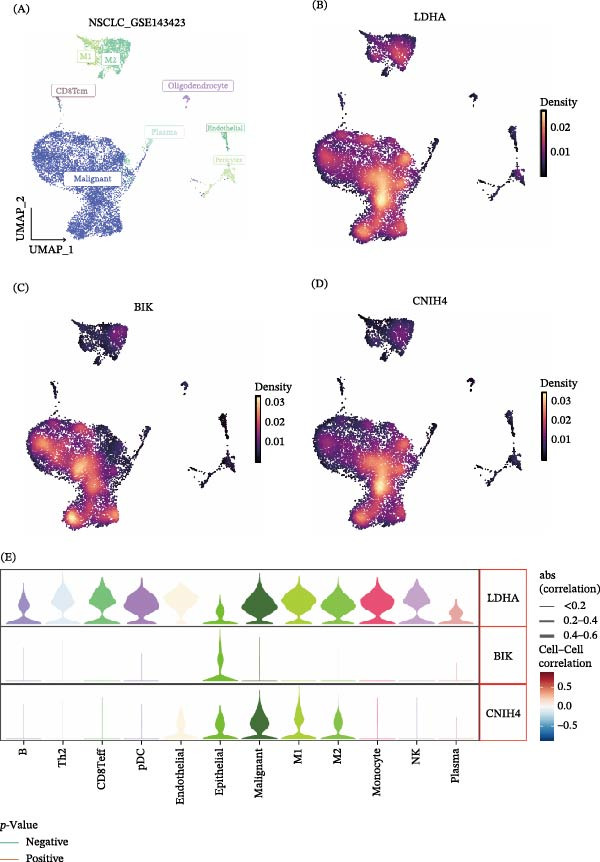

**Figure:**
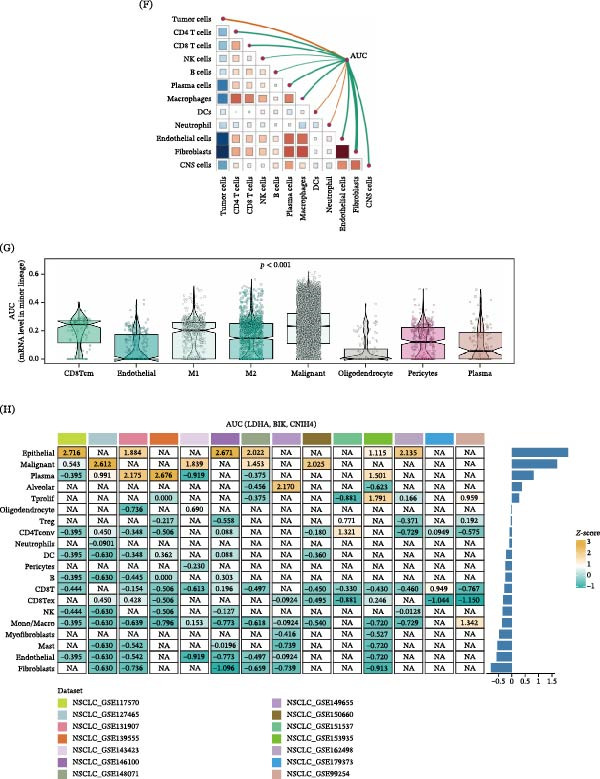

### 3.9. Screening of Potential Drugs and Molecular Docking

**Figure 10:**
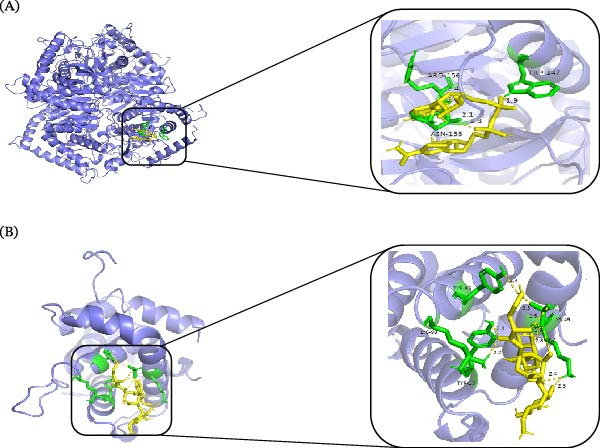
Protein‐drug molecular docking. (A) Molecular docking results of LDHA with compound tetrahydro‐NAD. (B) Molecular docking results of BIK with compound amikacin.

CNIH4 forecasted the compounds; however, the protein structures associated with the genes were unavailable in the PDB database. The molecular docking results indicated that LDHA established four hydrogen bonds with the residues ARG‐156, TRP‐147, and ASN‐155 of the compound tetrahydro‐NAD, resulting in a stable structure with a binding energy of −9.26 kcal/mol (Figure 10A). Additionally, BIK interacted with the compound amikacin, establishing 10 hydrogen bonds with TYR‐95, LYS‐49, and LYS‐98, resulting in a binding energy of −5.69 kcal/mol (Figure 10B), which suggests a strong affinity between them.

### 3.10. Clinical and In Vitro Functional Validation

**Figure 11:**
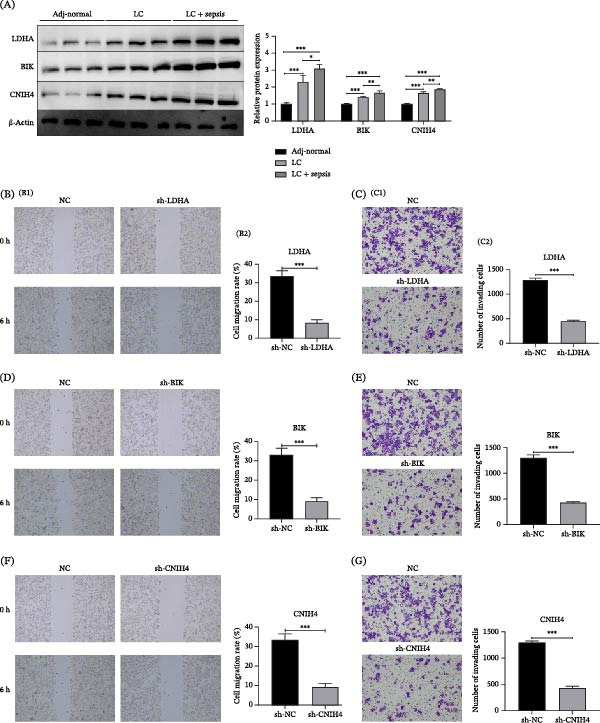
Protein expression levels of LDHA, BIK, and CNIH4 in clinical tissue specimens and their effects on the migration and invasion of lung cancer cells. (A) Western blot analysis and corresponding quantitative bar charts of LDHA, BIK, and CNIH4 protein expression levels in tumor‐adjacent normal tissues (Adj‐Normal), lung cancer tissues (LC), and lung cancer comorbid with sepsis tissues (LC+Sepsis). (B) Wound healing assay evaluating the effect of LDHA knockdown (sh‐LDHA) versus negative control (NC) on PC9 cell migration at 0 h and 6 h (B1), with the quantitative analysis of the cell migration rate shown in (B2). (C) Transwell invasion assay evaluating the effect of LDHA knockdown (sh‐LDHA) on the invasive ability of PC9 cells (C1), with the quantitative analysis of the number of invading cells shown in (C2). (D) Wound healing assay evaluating the effect of BIK knockdown (sh‐BIK) on PC9 cell migration at 0 h and 6 h, alongside the quantitative analysis of the cell migration rate. (E) Transwell invasion assay evaluating the effect of BIK knockdown (sh‐BIK) on PC9 cell invasion, alongside the quantitative analysis of the number of invading cells. (F) Wound healing assay evaluating the effect of CNIH4 knockdown (sh‐CNIH4) on PC9 cell migration at 0 h and 6 h, alongside the quantitative analysis of the cell migration rate. (G) Transwell invasion assay evaluating the effect of CNIH4 knockdown (sh‐CNIH4) on PC9 cell invasion, alongside the quantitative analysis of the number of invading cells. Data are presented as mean ± standard deviation;  ^∗^ indicates significance level ( ^∗^ *p* < 0.05,  ^∗∗^ *p* < 0.01,  ^∗∗∗^ *p* < 0.001).

To further validate our bioinformatics findings and explore the clinical significance of the identified hub genes, we collected clinical specimens, including Adj‐Normal, LC, and lung cancer comorbid with sepsis tissues (LC + Sepsis). Protein expression levels of LDHA, BIK, and CNIH4 were determined by western blot. The results showed that (Figure 11A), compared with the Adj‐Normal group, the protein expression levels of LDHA, BIK, and CNIH4 in the LC group were significantly increased, by 2.32 ± 0.37‐fold (*p* < 0.001), 1.42 ± 0.04‐fold (*p* < 0.001), and 1.67 ± 0.07‐fold (*p* < 0.001), respectively. Similarly, the protein levels in the LC + Sepsis group were also significantly elevated compared to the Adj‐Normal group, with increases of 3.11 ± 0.24‐fold (*p* < 0.001) for LDHA, 1.68 ± 0.11‐fold (*p* < 0.001) for BIK, and 1.88 ± 0.03‐fold (*p* < 0.001) for CNIH4. Notably, the expression levels of these three proteins were further significantly upregulated in the LC + Sepsis group compared with the LC group (LDHA: *p* < 0.05; BIK: *p* < 0.01; CNIH4: *p* < 0.001). These findings not only validated our initial predictions at the protein level but also confirmed the specific high‐expression profile of these three biomarkers during the pathological progression of lung cancer comorbid with sepsis.

To investigate the biological functions of these three hub genes in lung cancer progression, we performed in vitro functional experiments using the PC9 lung cancer cell line. We knocked down the expression of LDHA, BIK, and CNIH4, respectively, with cells transfected with negative control (NC) sequences serving as the control group.

Silencing of LDHA significantly inhibited cell migration and invasion. Compared with the NC group, the migration rate of PC9 cells in the sh‐LDHA group decreased from 33.55% ± 2.96% to 8.3% ± 1.69%, and the number of invading cells decreased from 1279.67 ± 48.6 to 449.67 ± 21.22 (all *p* < 0.001).

Silencing BIK also significantly suppressed cell migration and invasion. Compared with the NC group, the migration rate of PC9 cells in the sh‐BIK group decreased from 33.19% ± 3.29% to 9.22% ± 1.65%, and the number of invading cells decreased from 1300.00 ± 60.65 to 432.00 ± 8.89 (all *p* < 0.001).

Silencing CNIH4 significantly inhibited cell migration and invasion. Compared with the NC group, the migration rate of PC9 cells in the sh‐CNIH4 group decreased from 33.51% ± 3.1% to 9.24% ± 1.76%, and the number of invading cells decreased from 1296.67 ± 31.51 to 430.33 ± 33.08 (all *p* < 0.001).

In summary, results from both clinical specimens and in vitro experiments confirm the aberrant overexpression of LDHA, BIK, and CNIH4 in lung cancer comorbid with sepsis, demonstrating that they play critical roles in promoting the migration and invasion of lung cancer cells.

## 4. Discussion

Lung cancer is a significant cause of sepsis. Due to the distinctive architecture of the lungs that links to the external environment, lung cancer patients exhibit increased vulnerability to bacterial invasion, leading to lung infections that subsequently facilitate the onset of sepsis [23]. Lung cancer and sepsis exhibit analogous pathophysiological characteristics, including impairment of innate and adaptive immune cells [8, 24]. The primary cause of lung cancer‐induced immunosuppression is acquired immunological dysfunction resulting from cancer therapies, particularly chemotherapy and radiotherapy, which can reduce neutrophil counts and compromise their phagocytic capabilities [25]. A study in mice has demonstrated that lung cancer could trigger systemic T‐cell depletion and immunosuppression, thereby heightening vulnerability to sepsis [26].

The involvement of ERS in lung cancer and sepsis has been demonstrated in numerous research. For instance, ERS can stimulate the cellular inflammatory cascade and facilitate the onset and progression of lung cancer, sepsis, and other diseases [10, 11]. Research indicates that inhibiting a high level of mutated tumor protein p53 (TP53) can effectively induce ERS and suppress tumor metastasis and recurrence in lung cancer patients [27]; the ERS‐related protein TMEM173 can proficiently modulate the release of ER calcium in macrophages and monocytes, thus influencing the onset and progression of sepsis [15]. These results indicate that ERS may possess significant therapeutic potential in lung cancer and sepsis. Despite the partial clarification of the intricate processes of ERS, accumulating data indicates the significance of complex molecular interactions (such as autophagy‐ERS and autophagy‐apoptosis crosstalk) in governing the pathogenesis of lung cancer and sepsis [28, 29]. Nonetheless, the precise molecular processes of lung cancer comorbid with sepsis remain barely explained.

No research has yet investigated the role of ERS biomarkers in lung cancer comorbid with sepsis using bioinformatics and machine learning methods. We used a synthesis of bioinformatics analysis and machine learning models to identify diagnostic genes for lung cancer comorbid with sepsis, pinpointing three hub genes (LDHA, BIK, and CNIH4) as diagnostic biomarkers for this condition. Our research indicated that immune‐related processes are essential in lung cancer comorbid with sepsis. Eventually, by molecular docking, we identified the molecules tetrahydro‐NAD and amikacin as prospective therapeutics for the management of lung cancer comorbid with sepsis. The study identified possible indicators of ERS for diagnosing lung cancer comorbid with sepsis, offering novel options for the diagnosis and treatment of this condition.

LDHA is a crucial component of glucose metabolism and plays an important role in cellular metabolism mainly by catalyzing the conversion of pyruvate to lactate [30]. Kayser et al. [31] discovered that over 90% of lung tissues from NSCLC patients exhibited positive LDHA expression, while all non‐tumor lung tissues exhibited negative LDHA expression. Research by Li et al. [32] indicates that starvation‐induced autophagy modulates metabolic reprograming by facilitating Axin1 degradation and β‐catenin nuclear translocation, which in turn enhances the transcriptional expression of LDHA and the malignant progression of bladder cancer. Silencing the LDHA gene can significantly diminish lactate production by tumors, hence reactivating the immunological response of T cells and NK cells [33]. Additionally, research indicates that the inhibition of LDHA expression might significantly mitigate the Warburg effect and the inflammatory response in sepsis [34]. This study demonstrated a trend of downregulation in LDHA expression in both the lung cancer and sepsis groups. However, there is a paucity of research regarding the significance of LDHA in lung cancer comorbid with sepsis, and no studies have substantiated the importance of LDHA in this context. We hypothesize that LDHA may affect the course of lung cancer comorbid with sepsis through metabolic regulation, but further experimental research is required to validate this assertion.

BIK is a proapoptotic protein within the Bcl‐2 family, predominantly situated in the ER, and facilitates the advancement of lung cancer by apoptosis [35]. Research has demonstrated that the stimulation of the EZH2/BIK axis can effectively facilitate the onset of lung cancer [36]. BIK plays a pivotal role in the apoptosis of vascular endothelial cells by modulating calcium influx from the ER to the mitochondria [37]. Moreover, BIK participates in the onset and progression of lung cancer and sepsis via apoptosis‐related pathways. Inhibition of the ERK/Bcl‐2 signaling pathway can significantly retard tumor cell proliferation, thereby contributing to the treatment of lung cancer [38]; activation of the BDNF/TrkB signaling pathway can enhance autophagy and suppress apoptosis in sepsis‐associated encephalopathy, contributing to the treating of sepsis and its neurological complications [39]. Our studies indicated that BIK expression levels were reduced in both lung cancer and sepsis groups. Nevertheless, research on the interplay between BIK and lung cancer comorbid with sepsis is rare, and the potential influence of BIK on the evolution of lung cancer comorbid with sepsis through the modulation of ERS deserves further investigation.

CNIH4, a member of the cornichon family, plays a significant role in essential physiological and pathological processes, including neurotransmitter modulation, tumor immunological microenvironment, and inflammatory response [40]. CNIH4 is markedly overexpressed in liver hepatocellular carcinoma (LIHC) tissues and exhibits significant associations with the growth of gastric cancer cells [41]. Research has demonstrated [42] that elevated expression of CNIH4 in breast cancer correlates with distinct immunological subtypes characterized by an IFN‐γ‐dominated immune response. However, there are few studies directly examining the correlation between CNIH4 and sepsis. Since sepsis encompasses a broad spectrum of inflammatory responses and immune modulation, CNIH4 may play an indirect role in the physiological and pathological mechanisms of sepsis by influencing cell signaling and the production of inflammatory mediators. Therefore, a comprehensive investigation into the correlation between CNIH4 and lung cancer as well as sepsis is necessary.

The enrichment results indicated that these three hub genes are involved in significant pathogenic processes of lung cancer and sepsis via the Nod‐like receptor signaling pathway, MAPK signaling pathway, Wnt signaling pathway, et cetera. For instance, LDHA induces cardiomyocyte pyroptosis by facilitating NLRP3 lactylation, eventually exacerbating cardiac ischemia‐reperfusion injury [43]; inhibiting the LDHA/ROS/NLRP3 pathway can significantly mitigate renal tubular inflammation [44]. Furthermore, LDHA can proficiently modulate tumor‐macrophage symbiosis, consequently facilitating the onset and progression of glioblastoma [45]. However, the involvement of these hub genes in the critical mechanisms of lung cancer comorbid with sepsis via these immune cells require additional investigation.

The findings on immune infiltration indicate that cellular immune dysfunction, resulting from the interaction of various immune cells, may be the primary pathological mechanism underlying lung cancer comorbid with sepsis, in which LDHA serves as a vital gene facilitating the participation of plasma cells, monocytes, and other immune cells in this condition [46, 47]. Our findings suggest that the hub genes influence lung cancer‐sepsis comorbidity by modulating the functional state of specific immune subsets. Specifically, LDHA was positively correlated with plasma cells but negatively correlated with CD8+ T cells and monocytes in lung cancer. Given that LDHA‐mediated lactate production is known to blunt tumor immunosurveillance [48], its downregulation in our comorbid models may reflect a state of metabolic exhaustion that impairs the phagocytic and antigen‐presenting capabilities of monocytes, potentially heightening the susceptibility to secondary infections in sepsis [49]. Similarly, CNIH4’s positive correlation with neutrophils and negative correlation with CD8+ T cells in lung cancer indicate its potential role in regulating the recruitment of myeloid‐derived suppressor cells [50], which could further exacerbate the cytokine storm and systemic immunosuppression characteristic of sepsis [51]. Research indicates a consistent positive correlation between the abundance of tissue‐resident memory T cells and CD4+ T helper 1 cells, M1 macrophages, and resting dendritic cells within the lung cancer TME [52]; tumor‐infiltrating B cells are present at all stages of lung cancer and significantly influence tumor development, either promoting or inhibiting it [53]. Moreover, the manipulation of immunological receptors on mast cells and dendritic cells might efficiently facilitate neutrophil recruitment during sepsis [54]. These pieces of evidence offer robust support for our conclusions.

A previous study has investigated the potential involvement of immune‐related genes in lung cancer and sepsis [49, 50]; but our study concentrates on the significance of ESR‐related genes in lung cancer comorbid with sepsis. This innovative method uncovered significant insights into the influence of ERS on the pathophysiology of lung cancer comorbid with sepsis, a domain that has not been extensively explored in prior research. Our research eventually identified diagnostic markers for lung cancer comorbid with sepsis and forecasted possible treatment agents for this condition. All of this not only enhances comprehension of the molecular pathways between lung cancer and sepsis but also indicates an important pathway for future research.

Our bioinformatics research has discovered three hub genes related to ERS, which may significantly contribute to the progression of lung cancer comorbid with sepsis. However, our study has following limitations: (1) restricted sample size: the relatively small sample size may affect the precision of the results. Furthermore, the perfect AUC (1.000) observed in our diagnostic model is likely an artifact of the extremely small sample size of the external validation cohort (GSE118370, *n* = 12). Subsequent research must be performed on more extensive datasets to guarantee the validity of the findings. (2) Methodological limitations of in silico discovery: biologically, the microenvironment of a comorbidity is not a simple mathematical intersection of signatures from two separate diseases, which represents an inherent limitation of our initial computational discovery phase. (3) Specificity validation of generalized stress: while our in vitro and clinical tissue experiments validated the functional roles of these genes, the lack of cross‐disease “negative control” cohorts (e.g., noninflammatory metabolic datasets) limits our ability to completely distinguish this signature from a generalized cellular stress state. Future prospective validation across broader disease spectrums is required. (4) In vitro experimental design: while our in vitro functional assays confirmed the baseline oncogenic roles of these genes, they were conducted under standard conditions. Future studies must incorporate inflammatory stimuli (e.g., lipopolysaccharide or septic patient serum) to rigorously simulate the septic microenvironment and evaluate how these biomarkers modulate tumor cell behavior under dual inflammatory and malignant stress. (5) Potential confounding factors: this study exclusively employed the lung cancer and sepsis datasets from the GEO database and did not account for the influence of additional variables (such as diet, work and rest routine, age, gender, etc.) on the expression of the identified genes, which may introduce a certain extent of bias. (6) The complexity of disease mechanisms: both lung cancer and sepsis are multifactorial conditions characterized by intricate genetic and environmental interactions. Our research concentrated on a limited selection of genes, which may not comprehensively encompass all the molecular pathways implicated in disease development.

## 5. Conclusion

In summary, this study provides a novel integrated framework of machine learning and single‐cell analysis to bridge the gap between ERS and immune signaling in lung cancer comorbid with sepsis. Our major contribution is the identification and validation of LDHA, BIK, and CNIH4 as a stable diagnostic panel, exhibiting consistent downregulation in both conditions and strong cell‐type‐specific expression in malignant cells. Furthermore, our discovery of tetrahydro‐NAD and amikacin through molecular docking offers promising chemical scaffolds for multitarget therapy. Future research should prioritize the following: (i) functional validation using CRISPR‐Cas9 or gene knockdown models to elucidate the specific ERS‐immune crosstalk pathways and (ii) prospective clinical trials with larger cohorts to establish the clinical sensitivity of these biomarkers in peripheral blood samples.

## Funding

This study was funded by the Joint University‐Affiliated Hospital Scientific and Technological Innovation Fund of Guangzhou University of Chinese Medicine (Grant GZYFS2024G01).

## Conflicts of Interest

The authors declare no conflicts of interest.

## Supporting Information

Additional supporting information can be found online in the Supporting Information section.

## Supporting information


**Supporting Information 1** Table S1A: Differentially expressed genes in lung cancer.

**Supporting Information 2** Table S1B: Differentially expressed genes in sepsis.

**Supporting Information 3** Table S2A: WGCNA in lung cancer.

**Supporting Information 4** Table S2B: WGCNA in sepsis.

**Supporting Information 5** Table S3: Intersection gene GO enrichment analysis.

**Supporting Information 6** Table S4: Intersection gene KEGG enrichment analysis.

**Supporting Information 7** Table S5: Results of GSEA analysis of hub genes.

**Supporting Information 8** Table S6: Results of GSVA analysis of hub genes.

## Data Availability

The data provided in the study can be seen in the supporting information table in our submission document.

## References

[1] Bray F. , Laversanne M. , and Sung H. , et al.Global Cancer Statistics 2022: GLOBOCAN Estimates of Incidence and Mortality Worldwide for 36 Cancers in 185 countries, CA: A Cancer Journal for Clinicians. (2024) 74, no. 3, 229–263, 10.3322/caac.21834. 38572751

[2] Thai A. A. , Solomon B. J. , Sequist L. V. , Gainor J. F. , and Heist R. S. , Lung Cancer, The Lancet. (2021) 398, no. 10299, 535–554, 10.1016/S0140-6736(21)00312-3. 34273294

[3] Singer M. , Deutschman C. S. , and Seymour C. W. , et al.The Third International Consensus Definitions for Sepsis and Septic Shock (Sepsis-3), JAMA. (2016) 315, no. 8, 801–810, 10.1001/jama.2016.0287. 26903338 PMC4968574

[4] Fleischmann-Struzek C. , Mellhammar L. , and Rose N. , et al.Incidence and Mortality of Hospital- And ICU-Treated Sepsis: Results From an Updated and Expanded Systematic Review and Meta-Analysis, Intensive Care Medicine. (2020) 46, no. 8, 1552–1562, 10.1007/s00134-020-06151-x. 32572531 PMC7381468

[5] Tripathi A. K. and Srivastava Y. , The Overlapping Biology of Sepsis and Cancer and Therapeutic Implications, Biomedicines. (2025) 13, no. 6, 10.3390/biomedicines13061280, 1280. 40563999 PMC12189366

[6] Gan Z. , Zhang J. , and Huang J. , et al.In-Hospital Survival Characteristics and Predictive Model for Patients With Malignant Tumors and Sepsis, Frontiers in Medicine. (2026) 13, 10.3389/fmed.2026.1751311, 1751311. 41822889 PMC12975745

[7] Guo X. , Song J. , Liu M. , Ou X. , and Guo Y. , The Interplay Between the Tumor Microenvironment and Tumor-Derived Small Extracellular Vesicles in Cancer Development and Therapeutic Response, Cancer Biology & Therapy. (2024) 25, no. 1, 10.1080/15384047.2024.2356831, 2356831. 38767879 PMC11110713

[8] van der Poll T. , van de Veerdonk F. L. , Scicluna B. P. , and Netea M. G. , The Immunopathology of Sepsis and Potential Therapeutic Targets, Nature Reviews Immunology. (2017) 17, no. 7, 407–420, 10.1038/nri.2017.36. 28436424

[9] Hendershot L. M. , Buck T. M. , and Brodsky J. L. , The Essential Functions of Molecular Chaperones and Folding Enzymes in Maintaining Endoplasmic Reticulum Homeostasis, Journal of Molecular Biology. (2024) 436, no. 14, 10.1016/j.jmb.2023.168418, 168418. 38143019 PMC12015986

[10] Kim H. J. , Jeong J. S. , Kim S. R. , Park S. Y. , Chae H. J. , and Lee Y. C. , Inhibition of Endoplasmic Reticulum Stress Alleviates Lipopolysaccharide-Induced Lung Inflammation Through Modulation of NF-κB/HIF-1α Signaling Pathway, Scientific Reports. (2013) 3, no. 1, 10.1038/srep01142, 1142. 23359618 PMC3556596

[11] Fang C. , Weng T. , and Hu S. , et al.IFN-γ-Induced ER Stress Impairs Autophagy and Triggers Apoptosis in Lung Cancer Cells, OncoImmunology. (2021) 10, no. 1, 10.1080/2162402X.2021.1962591, 1962591. 34408924 PMC8366549

[12] Zeng T. , Zhou Y. , and Yu Y. , et al.rmMANF Prevents Sepsis-Associated Lung Injury via Inhibiting Endoplasmic Reticulum Stress-Induced Ferroptosis in Mice, International Immunopharmacology. (2023) 114, 10.1016/j.intimp.2022.109608, 109608. 36700778

[13] Chen X. and Cubillos-Ruiz J. R. , Endoplasmic Reticulum Stress Signals in the Tumour and Its Microenvironment, Nature Reviews Cancer. (2021) 21, no. 2, 71–88, 10.1038/s41568-020-00312-2. 33214692 PMC7927882

[14] Di Conza G. and Ho P.-C. , ER Stress Responses: An Emerging Modulator for Innate Immunity, Cells. (2020) 9, no. 3, 10.3390/cells9030695, 695. 32178254 PMC7140669

[15] Mandula J. K. , Chang S. , and Mohamed E. , et al.Ablation of the Endoplasmic Reticulum Stress Kinase PERK Induces Paraptosis and Type I Interferon to Promote Anti-Tumor T Cell Responses, Cancer Cell. (2022) 40, no. 10, 1145–1160, 10.1016/j.ccell.2022.08.016. 36150390 PMC9561067

[16] Zhang H. , Zeng L. , and Xie M. , et al.TMEM173 Drives Lethal Coagulation in Sepsis, Cell Host & Microbe. (2020) 27, no. 4, 556–570, 10.1016/j.chom.2020.02.004. 32142632 PMC7316085

[17] Ritchie M. E. , Phipson B. , and Wu D. , et al.Limma Powers Differential Expression Analyses for RNA-Sequencing and Microarray Studies, Nucleic Acids Research. (2015) 43, no. 7, 10.1093/nar/gkv007. PMC4402510 25605792

[18] Langfelder P. and Horvath S. , WGCNA: An R Package for Weighted Correlation Network Analysis, BMC Bioinformatics. (2008) 9, no. 1, 10.1186/1471-2105-9-559, 559. 19114008 PMC2631488

[19] Sanz H. , Valim C. , Vegas E. , Oller J. M. , and Reverter F. , SVM-RFE: Selection and Visualization of the Most Relevant Features Through Non-Linear Kernels, BMC Bioinformatics. (2018) 19, no. 1, 10.1186/s12859-018-2451-4, 432. 30453885 PMC6245920

[20] Hao M. , Wang Y. , and Bryant S. H. , An Efficient Algorithm Coupled With Synthetic Minority Over-Sampling Technique to Classify Imbalanced PubChem BioAssay Data, Analytica Chimica Acta. (2014) 806, 117–127, 10.1016/j.aca.2013.10.050. 24331047 PMC3884825

[21] de Vlaming R. and Groenen P. J. , The Current and Future Use of Ridge Regression for Prediction in Quantitative Genetics, BioMed Research International. (2015) 18, 10.1155/2015/143712, 143712. PMC4529984 26273586

[22] Yan J. , Pan Y. , and Chen C. , et al.Integrating Bioinformatics Analysis, Machine Learning, and Experimental Validation to Identify Pyroptosis-Related Genes in the Diagnosis of Sepsis Combined With Acute Liver Failure, Hereditas. (2025) 162, no. 1, 10.1186/s41065-025-00522-4, 153. 40781631 PMC12333193

[23] Meng X. Y. , Zhang H. X. , Mezei M. , and Cui M. , Molecular Docking: A Powerful Approach for Structure-Based Drug Discovery, Current Computer Aided-Drug Design. (2011) 7, no. 2, 146–157, 10.2174/157340911795677602. 21534921 PMC3151162

[24] Rosolem M. M. , Rabello L. S. , and Lisboa T. , et al.Critically Ill Patients With Cancer and Sepsis: Clinical Course and Prognostic Factors, Journal of Critical Care. (2012) 27, no. 3, 301–307, 10.1016/j.jcrc.2011.06.014. 21855281

[25] Williams J. C. , Ford M. L. , and Coopersmith C. M. , Cancer and Sepsis, Clinical Science. (2023) 137, no. 11, 881–893, 10.1042/CS20220713. 37314016 PMC10635282

[26] Mittal R. , Chen C. W. , and Lyons J. D. , et al.Murine Lung Cancer Induces Generalized T-Cell Exhaustion, Journal of Surgical Research. (2015) 195, no. 2, 541–549, 10.1016/j.jss.2015.02.004. 25748104 PMC4417390

[27] Bi Y. Y. , Chen Q. , Yang M. Y. , Xing L. , and Jiang H. L. , Nanoparticles Targeting Mutant p53 Overcome Chemoresistance and Tumor Recurrence in Non-Small Cell Lung Cancer, Nature Communications. (2024) 15, no. 1, 10.1038/s41467-024-47080-3, 2759. PMC10980692 38553451

[28] Liu G. , Pei F. , and Yang F. , et al.Role of Autophagy and Apoptosis in Non-Small-Cell Lung Cancer, International Journal of Molecular Sciences. (2017) 18, no. 2, 10.3390/ijms18020367, 367. 28208579 PMC5343902

[29] Chen H. G. , Han H. Z. , Li Y. , Yu Y. H. , and Xie K. L. , Hydrogen Alleviated Organ Injury and Dysfunction in Sepsis: The Role of Cross-Talk Between Autophagy and Endoplasmic Reticulum Stress: Experimental Research, International Immunopharmacology. (2020) 78, 10.1016/j.intimp.2019.106049, 106049. 31830624

[30] Urbańska K. and Orzechowski A. , Unappreciated Role of LDHA and LDHB to Control Apoptosis and Autophagy in Tumor Cells, International Journal of Molecular Sciences. (2019) 20, no. 9, 10.3390/ijms20092085, 2085. 31035592 PMC6539221

[31] Kayser G. , Kassem A. , and Sienel W. , et al.Lactate-Dehydrogenase 5 is Overexpressed in Non-Small Cell Lung Cancer and Correlates With the Expression of the Transketolase-Like Protein 1, Diagnostic Pathology. (2010) 5, no. 1, 10.1186/1746-1596-5-22, 22. 20385008 PMC2861018

[32] Li T. , Tong H. , and Yin H. , et al.Starvation Induced Autophagy Promotes the Progression of Bladder Cancer by LDHA Mediated Metabolic Reprogramming, Cancer Cell International. (2021) 21, no. 1, 10.1186/s12935-021-02303-1, 597. 34743698 PMC8573950

[33] Brand A. , Singer K. , and Koehl G. E. , et al.LDHA-Associated Lactic Acid Production Blunts Tumor Immunosurveillance by T and NK Cells, Cell Metabolism. (2016) 24, no. 5, 657–671, 10.1016/j.cmet.2016.08.011. 27641098

[34] Zhang Q. , Luo P. , and Xia F. , et al.Capsaicin Ameliorates Inflammation in a TRPV1-Independent Mechanism by Inhibiting PKM2-LDHA-Mediated Warburg Effect in Sepsis, Cell Chemical Biology. (2022) 29, no. 8, 1248–1259, 10.1016/j.chembiol.2022.06.011. 35858615

[35] Mebratu Y. A. and Tesfaigzi Y. , Does the BCL-2 Family Member BIK Control Lung Carcinogenesis?, Molecular & Cellular Oncology. (2018) 5, no. 4, 10.1080/23723556.2018.1435182. PMC6150014 30250907

[36] Yuan C. , Ding Y. , and Zhuang Y. , et al.Copy Number Amplification-Activated Long Non-Coding RNA LINC00662 Epigenetically Inhibits BIK by Interacting With EZH2 to Regulate Tumorigenesis in Non-Small Cell Lung Cancer, Journal of Cancer. (2022) 13, no. 5, 1640–1651, 10.7150/jca.69210. 35371316 PMC8965125

[37] Zhang P. , Yan X. , and Zhang X. , et al.TMEM215 Prevents Endothelial Cell Apoptosis in Vessel Regression by Blunting BIK-Regulated ER-to-Mitochondrial Ca Influx, Circulation Research. (2023) 133, no. 9, 739–757, 10.1161/CIRCRESAHA.123.322686. 37750320

[38] Gong Q. , Deng J. , and Zhang L. , et al.Targeted Silencing of TEM8 Suppresses Non-Small Cell Lung Cancer Tumor Growth via the ERK/Bcl-2 Signaling Pathway, Molecular Medicine Reports. (2021) 24, no. 2, 10.3892/mmr.2021.12234, 12234. PMC8240451 34165155

[39] Gao L. L. , Wang Z. H. , Mu Y. H. , Liu Z. L. , and Pang L. , Emodin Promotes Autophagy and Prevents Apoptosis in Sepsis-Associated Encephalopathy Through Activating BDNF/TrkB Signaling, Pathobiology. (2022) 89, no. 3, 135–145, 10.1159/000520281. 34872094 PMC9227694

[40] Shanks N. F. , Cais O. , and Maruo T. , et al.Molecular Dissection of the Interaction Between the AMPA Receptor and Cornichon Homolog-3, Journal of Neuroscience. (2014) 34, no. 36, 12104–12120, 10.1523/JNEUROSCI.0595-14.2014. 25186755 PMC4152608

[41] Zhang H. , Lin Y. , Zhuang M. , Zhu L. , Dai Y. , and Lin M. , Screening and Identification of CNIH4 Gene Associated With Cell Proliferation in Gastric Cancer Based on a Large-Scale CRISPR-Cas9 Screening Database DepMap, Gene. (2023) 850, 10.1016/j.gene.2022.146961, 146961. 36220450

[42] Xu Y. , Lai Z. , and Li C. , Deciphering the Role of CNIH4 in Pan-Cancer Landscapes and Its Significance in Breast Cancer Progression, Frontiers in Genetics. (2025) 16, 10.3389/fgene.2025.1536620, 1536620. 40051704 PMC11882561

[43] Fang L. , Yu Z. , Qian X. , Fang H. , and Wang Y. , LDHA Exacerbates Myocardial Ischemia-Reperfusion Injury Through Inducing NLRP3 Lactylation, BMC Cardiovascular Disorders. (2024) 24, no. 1, 10.1186/s12872-024-04251-w, 651. 39548367 PMC11568565

[44] Ouyang J. , Wang H. , Gan Y. , and Huang J. , Uric Acid Mediates Kidney Tubular Inflammation Through the LDHA/ROS/NLRP3 Pathway, Clinical and Experimental Hypertension. (2024) 46, no. 1, 10.1080/10641963.2024.2424834, 2424834. 39488824

[45] Khan F. , Lin Y. , and Ali H. , et al.Lactate Dehydrogenase A Regulates Tumor-Macrophage Symbiosis to Promote Glioblastoma Progression, Nature Communications. (2024) 15, no. 1, 10.1038/s41467-024-46193-z, 1987. PMC10914854 38443336

[46] Zhang W. , Chen Q. , and Li Y. , et al.Integrated Bulk and Single-Cell Transcriptomics to Develop an Efferocytosis-Related Prognostic Model for Lung Adenocarcinoma and Validate the Key Gene LDHA, European Journal of Medical Research. (2026) 31, no. 1, 10.1186/s40001-025-03622-z, 49. PMC12798036 41345972

[47] Pan D. , Zou B. , Wang Y. , and Yu L. , LDHA Associated With Telomerase-Related Genes Promotes Tumorigenesis and Progression of Lung Adenocarcinoma, Scientific Reports. (2025) 15, no. 1, 10.1038/s41598-025-25626-9, 41677. 41286077 PMC12644902

[48] Zhang T. , Chen L. , and Kueth G. , et al.Lactate’s Impact on Immune Cells in Sepsis: Unraveling the Complex Interplay, Frontiers in Immunology. (2024) 15, 10.3389/fimmu.2024.1483400, 1483400. 39372401 PMC11449721

[49] Hattori S. , Saggar R. , and Heidinger E. , et al.Advances in Ultrasound-Guided Surgery and Artificial Intelligence Applications in Musculoskeletal Diseases, Diagnostics. (2024) 14, no. 18, 2008. 39335687 10.3390/diagnostics14182008 PMC11431371

[50] Verbov J. , Topical Tartrazine as an Unusual Cause of Nail Staining, British Journal of Dermatology. (1985) 112, no. 6, 10.1111/j.1365-2133.1985.tb02348.x, 729. 4005170

[51] Qin S. , Kang Z. , and Wu Q. , et al.Targeting Myeloid-Derived Suppressor Cells in Sepsis: Pathophysiology, Duality, and Therapeutic Frontiers, Shock. (2026) 65, no. 5, 761. 41823710 10.1097/SHK.0000000000002817 PMC13132074

[52] Shen A. , Garrett A. , and Chao C. C. , et al.A Comprehensive Meta-Analysis of Tissue Resident Memory T Cells and Their Roles in Shaping Immune Microenvironment and Patient Prognosis in Non-Small Cell Lung Cancer, Frontiers in Immunology. (2024) 15, 10.3389/fimmu.2024.1416751, 1416751. 39040095 PMC11260734

[53] Wang S. S. , Liu W. , Ly D. , Xu H. , Qu L. , and Zhang L. , Tumor-Infiltrating B Cells: Their Role and Application in Anti-Tumor Immunity in Lung Cancer, Cellular & Molecular Immunology. (2019) 16, no. 1, 6–18, 10.1038/s41423-018-0027-x. 29628498 PMC6318290

[54] Udayanga K. G. , Nakamura Y. , Nakahashi-Oda C. , and Shibuya A. , Immunoreceptor CD300a on Mast Cells and Dendritic Cells Regulates Neutrophil Recruitment in a Murine Model of Sepsis, International Immunology. (2016) 28, no. 12, 611–615, 10.1093/intimm/dxw047. 27836913

